# Efficient gene editing through an intronic selection marker in cells

**DOI:** 10.1007/s00018-022-04152-1

**Published:** 2022-01-31

**Authors:** Shang Wang, Yuqing Li, Li Zhong, Kai Wu, Ruhua Zhang, Tiebang Kang, Song Wu, Yuanzhong Wu

**Affiliations:** 1 State Key Laboratory of Oncology in South China, Collaborative Innovation Center for Cancer Medicine, Department of Experimental Research, Sun Yat-Sen University Cancer Center, Guangzhou, 510060 China; 2 Institute of Urology, The Third Affiliated Hospital of Shenzhen University, Shenzhen, 518000 China; 3 Teaching Center of Shenzhen Luohu Hospital, Shantou University Medical College, Shantou, 515000 China; 4 Center of Digestive Diseases, The Seventh Affiliated Hospital of Sun Yat-Sen University, Shenzhen, 518107 China; 5 Scientific Research Center, The Seventh Affiliated Hospital of Sun Yat-Sen University, Shenzhen, 518107 China; 6 Department of Urology, South China Hospital of Shenzhen University, Shenzhen, 518000 China

**Keywords:** Gene editing, HDR, Intron reporter, FACS, Conversion tract

## Abstract

**Background:**

Gene editing technology has provided researchers with the ability to modify genome sequences in almost all eukaryotes. Gene-edited cell lines are being used with increasing frequency in both bench research and targeted therapy. However, despite the great importance and universality of gene editing, the efficiency of homology-directed DNA repair (HDR) is too low, and base editors (BEs) cannot accomplish desired indel editing tasks.

**Results and discussion:**

Our group has improved HDR gene editing technology to indicate DNA variation with an independent selection marker using an HDR strategy, which we named Gene Editing through an Intronic Selection marker (GEIS). GEIS uses a simple process to avoid nonhomologous end joining (NHEJ)-mediated false-positive effects and achieves a DsRed positive rate as high as 87.5% after two rounds of fluorescence-activated cell sorter (FACS) selection without disturbing endogenous gene splicing and expression. We re-examined the correlation of the conversion tract and efficiency, and our data suggest that GEIS has the potential to edit approximately 97% of gene editing targets in human and mouse cells. The results of further comprehensive analysis suggest that the strategy may be useful for introducing multiple DNA variations in cells.

**Supplementary Information:**

The online version contains supplementary material available at 10.1007/s00018-022-04152-1.

## Background

Genetic mutations cause human diseases, including cancers and heritable disorders. Therefore, gene editing technologies that can easily correct or generate mutations are critical for clinical applications and bench research, respectively [1]. Prokaryotes-derived CRISPR-Cas systems mediate target DNA cleavage guided by protospacer adjacent motif (PAM) and sgRNAs that form the DNA-RNA heteroduplex with the target genome DNA. Cas12 and Cas9 have been widely applied for eukaryotic genome editing because of their high efficiency in generating DNA double-strand breaks (DSBs), after which the DNA is repaired. Despite the potential for precise base editing by DNA repair, such as homology-directed repair (HDR) and nonhomologous end joining (NHEJ) [2], the efficiency of these methods varies dramatically in different cell lines [3, 4].

Recently, an alternative gene editing strategy was developed that uses dead Cas9 or Cas9 nickase to target DNA via sgRNA and recruits base deaminase domains to accomplish C-to-T base conversions (with a cytosine base editor, CBE) or A-to-G base conversions (with an adenine base editor, ABE) without introducing DSBs. Although the efficiency of this strategy can reach as high as 60%, its off-target effects, inability to accomplish A-to-C, A-to-T, G-to-C or G-to-T conversion [5], and bystander effect might inhibit its application [6, 7].

Introns are transcribed together with exons as pre-mRNA but are spliced by the spliceosome complex so that mature mRNA does not contain intronic sequences [8]. Introns are frequently used as targets for HDR genome editing strategies [9, 10]. To assist in the retrieval of successfully repaired clones, the target gene coding sequence (CDS) can be tagged with a fluorescent protein at the N- or C-terminus as a selectable marker [11]. To remove these tags, seamless repair using piggyBac or sleeping beauty is available [12, 13]. However, this kind of selection can be used only when the target sites are near the terminus, and it changes the open reading frame (ORF), which may have unpredictable negative effects on genetic regulation. Furthermore, the marker is driven by the endogenous promoter, which might be too weak to make a difference for selection.

To solve the shortcomings of the current gene editing methods, we established an efficient gene editing system based on HDR-mediated intronic fluorescent protein insertion without disruption of endogenous gene splicing and expression, which we named Gene Editing through an Intronic Selection marker (GEIS). This strategy avoids donor DNA-mediated false positive cell clones and produces as many as 87.5% gene-edited cells in our tested loci. The results of further studies reveal its strong potential for use in 97% of exon editing applications and for multiple mutation introduction.

## Results

### The GEIS workflow generates *RELA*/*p65* S276C HEK293T cells within 1 month

**Fig. 1:**
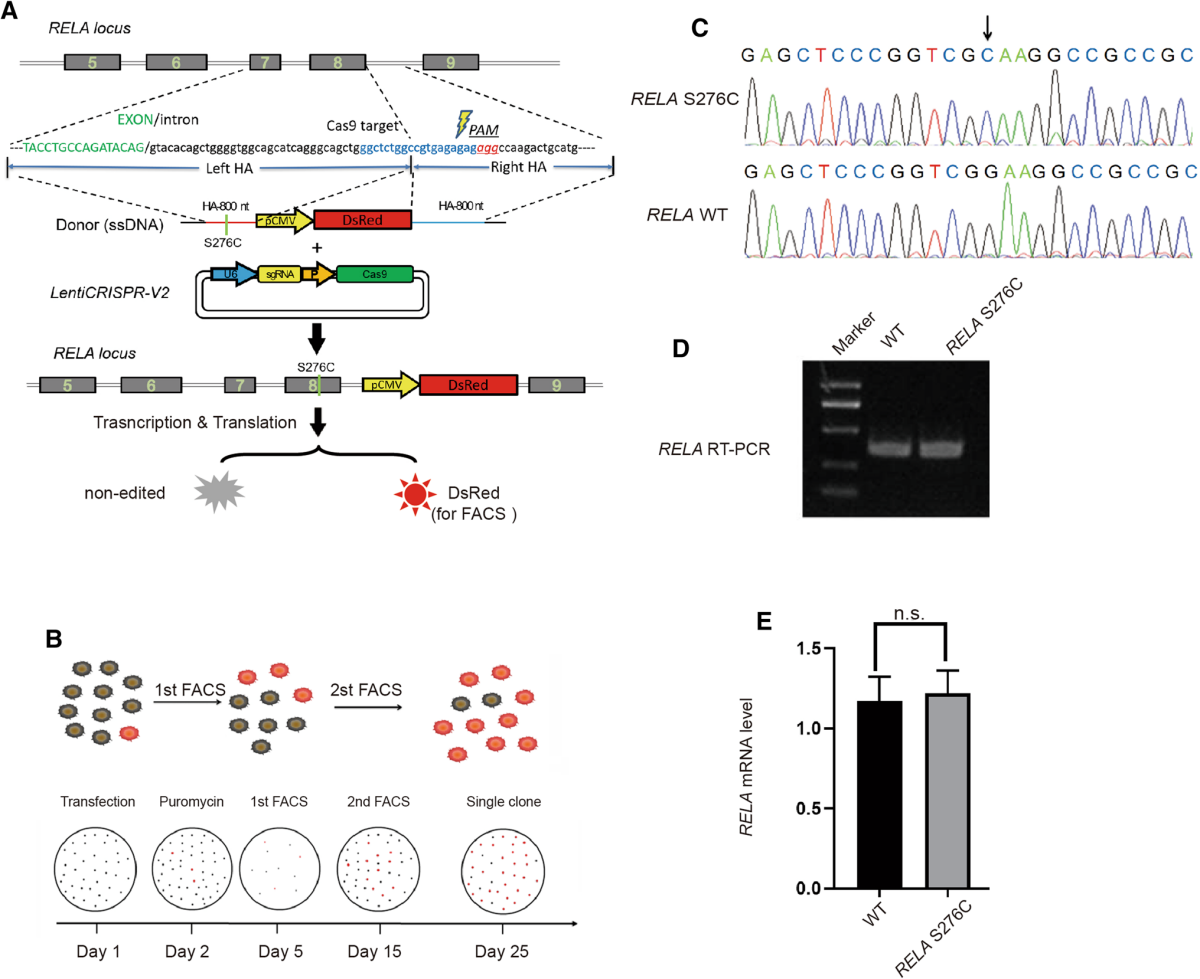
**A** Schematic view of GEIS to introduce the S276C mutation into the *RELA* locus in HEK293T cells. LentiCRISPR-V2 is used to generate DSBs in intron 8 and donor ssDNA as a template to introduce mutations by HDR. **B** Workflow of GEIS, with two rounds of FACS to enrich and fractionate individual DsRed-positive cells. **C** Sanger sequencing of *RELA* genomic DNA sequences derived from WT- and GEIS-treated *RELA* S276C gene-edited HEK293T cells. **D** RT–PCR of cDNA from WT and S276C HEK293T cells. No alternative variants were found. **E** Relative expression of *RELA* in WT and S276C HEK293T cells. No significant (n. s.) change in *RELA* expression was detected

p65 is a REL-associated protein involved in NF-κB heterodimer formation, nuclear translocation, and downstream gene transactivation [14]. We applied GEIS to generate the S276C mutation in *RELA*/p65. A LentiCRISPR-v2 plasmid carrying sgRNA targeting intron 8 of the *RELA* gene was used to generate DSBs. To avoid disrupting RNA splicing, we did not target the splice site. The donor DNA template contained a cytomegalovirus (CMV) promoter-driven DsRed-expressing cassette between the left and right homology arms (HAs), while the desired S276C mutation was located on the left arm (Fig. 1A). The LentiCRISPR-v2 plasmid and donor DNA were cotransfected into HEK293T cells for 24 h, and then puromycin selection was conducted for 72 h to kill nontransfected cells. The surviving cells were subjected to FACS to fractionate the DsRed-positive cells. To increase the selection efficiency, a second round of FACS was performed. The sorted cells were seeded into 96-well plates for single-cell clone growth. We obtained positive cell clones with the S276C mutation within 1 month with this workflow (Fig. 1B, C). Reverse transcription PCR (RT–PCR) and quantitative PCR (qPCR) showed that the inclusion of the CMV-DsRed cassette in the intron neither disturbed the splicing of the two adjacent exons nor affected mRNA transcription (Fig. 1D, E).

### HDR with an ssDNA template reduces the production of false-positive cell clones

**Fig. 2:**
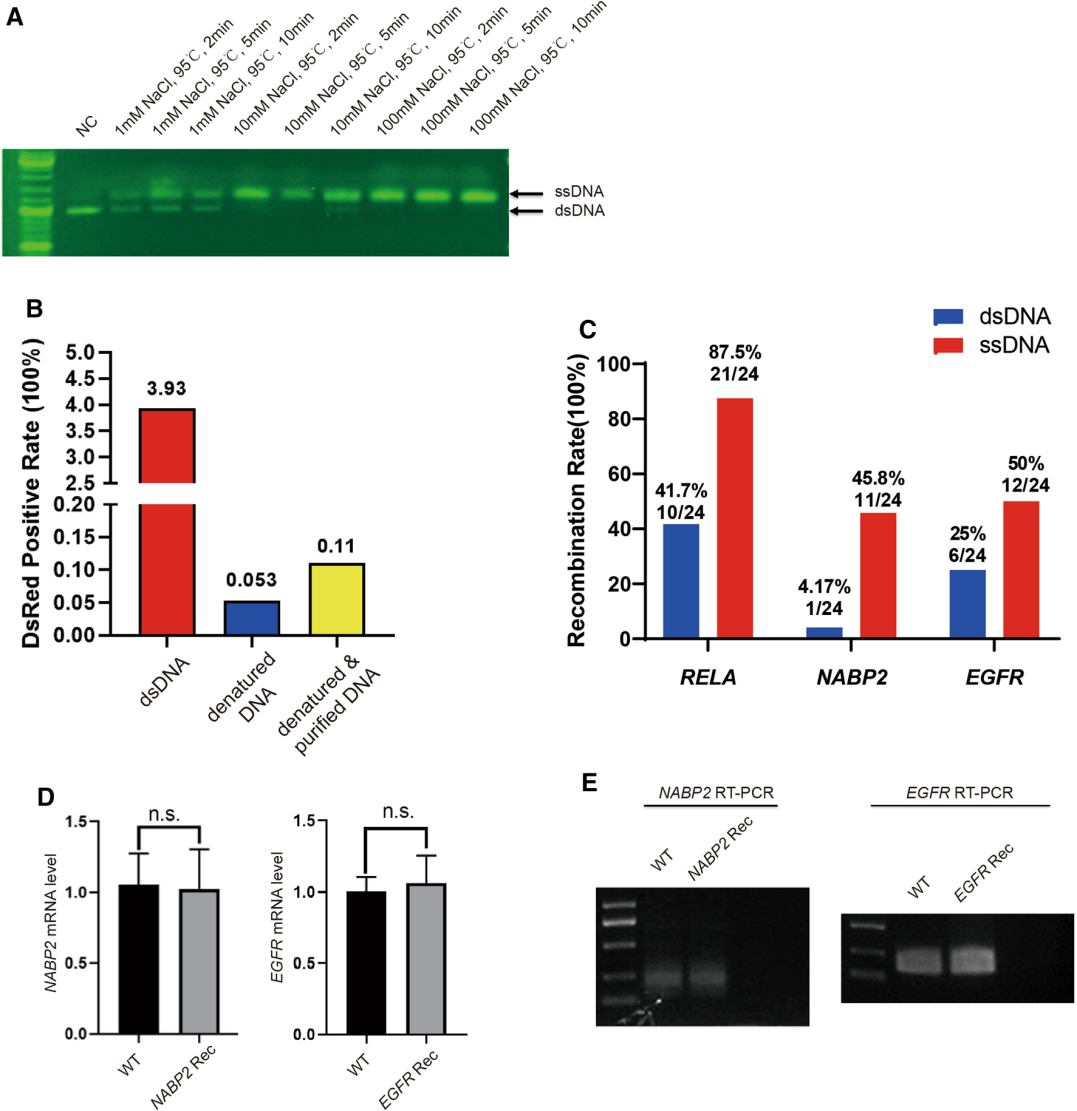
**A** Agarose gel electrophoresis of denaturing dsDNA under the indicated conditions. **B** Percentage of DsRed-positive cells from 1 μg of dsDNA-, ssDNA(denatured)- or ssDNA (denatured and purified)-transfected HEK293T cells determined by FACS. **C** HDR efficiency of *RELA*, *NABP2* and *EGFR* using GEIS using dsDNA (nondenatured) or ssDNA (denatured) as donor DNA. **D**. qPCR of *NABP2* and *EGFR* in WT and GEIS-recombined (Rec) cells. Data are the mean ± s.d. of *n* = 3 biological independent experiments. No significant (n. s.) variation was found by Student’s t-test for either *NABP2* or *EGFR* mutants. **E** Agarose gel electrophoresis of RT–PCR products of NABP2 and EGFR in WT- and GEIS-modified cells. No alternative variant was found

In this strategy, the use of dsDNA as donor DNA produces false-positive cell clones via direct transcription and translation or via random integration into the genome through canonical NHEJ (c-NHEJ) [15]. Recent studies have demonstrated that ssDNA donors show superior performance compared to dsDNA donors in mammalian systems by reducing the probability of NHEJ [16]. To effectively obtain ssDNA sequences as large as 5000 nt, we denatured the dsDNA from PCR at 95 °C and with 100 mM NaCl for 10 min (Fig. 2A). Transfection of dsDNA or ssDNA without CRISPR–Cas9 demonstrated that a single-stranded CMV-DsRed donor led to significantly lower fluorescence intensity than a double-stranded donor. Considering that NaCl might influence transfection, we purified DNA. The purification of ssDNA resulted in a slight increase in DsRed-positive cells (Figs. 2B, S1A, B). Based on the above results, we speculated that the use of an ssDNA donor would increase the true-positive rate of FACS-enriched DsRed-expressing cells. As shown in Fig. 2C, with ssDNA as a donor cotransfected with CRISPR–Cas9, the recombination rate for *RELA* reached 87.5% (21 out of 24), while with dsDNA, it was only 41.7% (10 out of 24) after two rounds of sorting. Elevated recombination rates were also observed at the *NABP2* and *EGFR* loci, and no abnormal splicing or mRNA level changes were detected (Fig. 2C–E).

### Conversion tract length influence gene editing efficiency

Despite the efficient selection of successfully recombined cells, GEIS still exhibits a low efficiency when the conversion tract is too long. Because the CMV-DsRed cassette must be located in an intron to avoid disrupting endogenous gene splicing and expression, the sgRNA target site should usually be intronic, but the expected conversion site is usually exonic. The distance from the DSB to the conversion site (conversion tract) affects the efficiency [17].

**Fig. 3:**
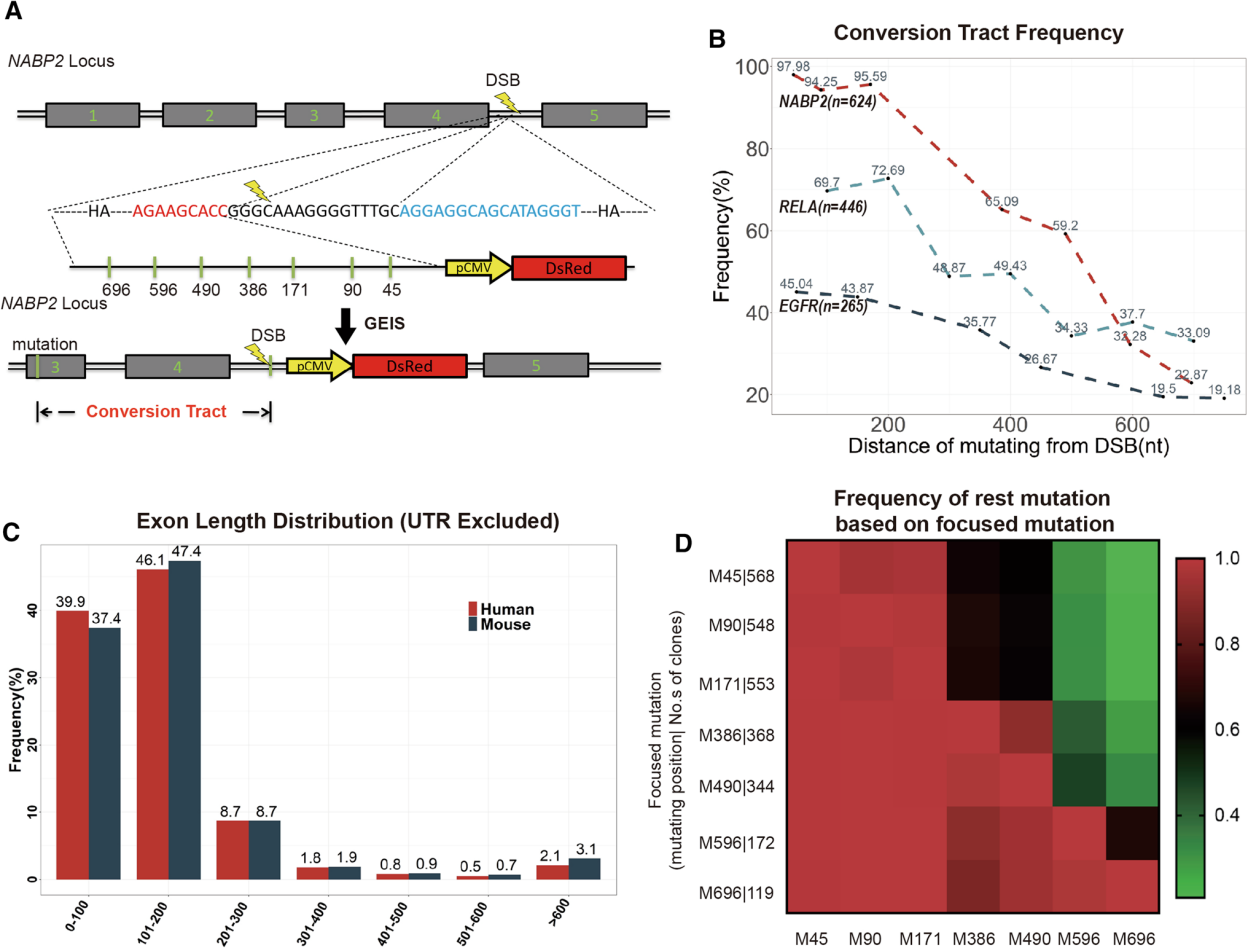
**A** Schematic overview of the genome editing conversion tract experiments in the *NABP2* locus: seven different DNA variations were included in the left HA of donor DNA in GEIS to test how many of these variations could be introduced in the genome locus of *NABP2*. **B** Percentage of DNA variations from the workflow of **A** in *NABP2*, *RELA* and *EGFR* that were introduced in the genome based on PCR amplicons. **C** Calculation of exon lengths of all human and mouse exons (UTR excluded). **D** Calculation of the selection efficiency of each variation indicated in **B** from Sanger sequencing of 624 clones that were PCR amplified from the GEIS-edited cell mix. When alteration A (vertical axis, M stands for mutated position, numbers stand for numbers of mutated clones) occurs, the percentage of the remaining alterations (horizontal axis, M stands for mutated position) is presented on a heatmap

To estimate the influence of conversion tract length on efficiency, we first evaluated the HA length required for efficient insertion of the selection cassette into the intron. Using the *EGFR* locus as an example, we designed a series of donors with 250, 500, 800 and 1000 nt HAs. We found that HAs longer than 500 nt were necessary for recombination at this locus (Fig. S2 A, B). Next, we designed donor DNA with a left HA (800 nt) containing nucleotide variations 45, 90, 171, 386, 490, 596 and 696 nt away from the DSB site for GEIS of *NABP2* (Fig. 3A). The genomic DNA of the GEIS-processed cell group was PCR-amplified with the forward primer located outside the left HA on the genome and the reverse primer at the DsRed cassette. The PCR product was cloned into pLV-MCS-puro-Green for Sanger sequencing. A total of 624 amplicons were sequenced, and the conversion efficiency was calculated. We repeated this workflow in *RELA* and *EGFR* recombination to test more loci, which introduce nucleotide switches, insertions and deletions (sequences are provided in the supplementary material). A total of 265 amplicons of *EGFR* and 446 amplicons of *RELA* were sequenced and calculated. In general, the conversion efficiency decreased as the tract became further away from the DSB. Although the efficiency varied largely in different loci, the conversion efficiency remained at 35% when the tract was shorter than 300 bp in the tested loci (Fig. 3B). We picked single-cell clones from *RELA-* and *EGFR*-edited cells for Sanger sequencing to exclude the effect of false amplicons generated by the possible overlap-extension process in PCR amplification. The results were highly consistent with those of amplicons (Fig. S2C). Previous research based on 80 cell clones reported an efficiency of only 20% when the tract was 200 bp long [17]. The extremely low efficiency might result from the DNA locus. Despite the variations, the three loci in this study showed far higher efficiencies than previously reported.

Because introns adjacent to the target exon on both the left and right sides are available for GEIS DSB generation, a nearer intron can always be found for the exonic editing site for GEIS, which needs less than half of the exon length as the conversion tract. For example, the conversion tract of 300 bp indicated that GEIS has at least a 35% probability of generating mutations for exons as large as 600 bp in the locus (Fig. S2D).

Furthermore, we investigated the requirement of conversion lengths in gene editing tasks. To assess the applicability of GEIS in the human and mouse genomes, we analyzed the distributions of exon length in these two species from the Consensus CDS (CCDS) Project (Fig. S2E) [18–20]. Most exons longer than 600 bp were the first or last exons, which contain long 5′ or 3′ untranslated regions (UTRs); however, DSBs can still be introduced by sgRNA in the first or last intron. When we excluded the UTRs and reanalyzed the distribution of exon lengths, only approximately 3% of exons had lengths longer than 600 bp **(**Fig. 3C**)**. Based on the conversion tract analysis from the *NABP2, RELA and EGFR loci*, we speculate that GEIS might be able to edit 97% of gene targets with relatively high efficiency.

### GEIS has the potential to introduce multiple DNA variations

To evaluate the possibility of introducing multiple genome alterations in one GEIS reaction, we analyzed the mutation distributions in each of the 624 *NABP2* amplicons. A heatmap was created to show the percentage of alterations that occurred at the remaining sites (horizontal axis) when an alteration occurred at the indicated site (vertical axis) (Fig. 3D). According to the map, mutations at a further site largely indicated successful editing of the nearer site, and genome editing showed a high extent of linkage rather than independence, indicating that multiple genome alterations can be introduced in one GEIS reaction.

## Discussion

Here, we have developed a universal and efficient HDR-based gene editing strategy in cell lines. Rather than tagging a fluorescent protein to the target gene ORF for FACS selection, we chose to insert a pCMV-driven DsRed selection marker into introns so that the selection marker did not influence target gene expression or splicing when indicating the desired DNA variations for FACS selection. To eliminate the possibility of false-positive cell clone generation by pCMV-driven DsRed-containing donor DNA and random integration of the DNA into the genome via NHEJ, we used ssDNA as the donor [21, 22]. To obtain bulk ssDNA sequences as large as 5000 nt [23], we simply denatured dsDNA at a high temperature in a certain concentration of salt, which is widely applied for all kinds of PCR [24, 25]. Given that an editing target that is too far away from an intron might result in failure to introduce a DNA variation, we explored the editing efficiency based on the conversion tract length, and the results are of great value for other gene editing strategies [26–28]. Considering these results and the analysis of the exon length distribution in humans and mice, we speculate that GEIS can accomplish nearly 97% of exon editing tasks at relatively high efficiency. In addition, GEIS’s ability to introduce multiple DNA variations was also assessed in this study. Such an ability has strong potential for use in the development of novel features based on a directed molecular evolution strategy [29, 30]. Overall, we have introduced a developed gene editing strategy and have described its risks as well as methods to avoid those risks. Our data can also be used in other gene editing applications and can support the use of novel strategies for specific editing tasks.

NHEJ and HDR are two ways to repair DNA after DSBs. The choice is determined by a series of factors, including cell cycle regulation [31] and chromatin context [32]. On the other hand, DSBs could arise during DNA replication, and NHEJ could randomly incorporate dsDNA into the genome in the DNA repair process [33]. Methods to impair NHEJ are useful to increase HDR efficiency [34, 35]. NHEJ inhibitors such as SCR7 increase the HDR product and knocking down SHROOM1 can increase HDR efficiency by 4.7-fold [36]. In this study, both NHEJ and HDR could generate DsRed-positive cells, but only HDR products were desired.

The conversion tract describes the distance of mutation and the DSB in donor DNA [37], which is important in HDR-mediated gene editing. If the conversion tract is too short, the chances of introducing desired DNA alterations are low. The conversion efficiency varies largely in different reports. Previous studies also reported only 20% when the tract was 200 bp [17]. In plants, the efficacy could remain at 80% when the tract is 600 bp [38]. In this study, we measured the conversion tract at three different loci, and the efficiency was as high as 65% for *NABP2* when the tract was 400 bp and 30% for *EGFR*. The efficiency could be influenced by the expression of *Pif1*, *Sgs1* and *Blm* [28, 39]. However, these factors cannot explain the difference in different loci in the same cell lines. More factors that influence conversion efficiencies are not yet clear.

This strategy is unlike intronic insertion knock-in HDR strategies, which are restricted to editing of bases near the stop or start codons for tagging markers that must be located at the N- or C-terminus of a CDS. The independent intronic marker strategy untethers the HDR gene editing strategy, allowing it to be used to solve editing problems in any exon in cells. Although similar intron targeting strategies have been reported, few of them eliminate the burden of in-frame marker tagging [10, 40, 41]. This strategy leaves a DsRed cassette in the genome that may contradict other experiments. To overcome this defect, if needed, we can turn to a scareless gene editing strategy [42] that employs an additional process of HDR to remove the DNA imprint in introns.

## Methods

### Plasmid and donor DNA

DSBs were generated by CRISPR–Cas9 technology with LentiCRISPR-V2 (Addgene, #98290) carrying the indicated intron-targeting sgRNAs (RELA: GGCUCUGUGCCGUGAGAGAG, NABP2: GGGCAAAGGGGUUUGCAAGG, EGFR: GCCAGCAUUUUCCUGACACC). *Important: sgRNAs should avoid targeting the GU-AG at the intron–exon boundaries and the pyrimidines required for RNA splicing!* The pDonor-GEIS plasmid was used as the framework for preparing the donor DNA. According to the sequence, two EcoRV sites were located adjacent to the pCMV-driving DsRed cassette for HA cloning. *Important: donor DNA should contain no terminators. *HAs were PCR amplified from HEK293T genomic DNA and inserted into the plasmid using a Gibson assembly cloning strategy. Mutations in HAs were generated by mutation- or truncation-containing primers using an overlap extension PCR strategy.

Donor DNA was generated from preprepared pDonor-GEIS (*RELA*, *NABP2*, and *EGFR*) by PCR using a pair of universal primers: Donor-F: TGTGGTGGAATTCTGCAGAT and Donor-R: GCGGCCGCCACTGTGCTGGAT. PCR was carried out in 28 cycles on an Eppendorf thermocycler with denaturation at 94 °C for 15 s, annealing at 58 °C for 15 s, and extension at 72 °C for 3 min using PrimeStar (TaKaRa, Japan). PCR products were purified using an Ultra-Sep Gel Extraction Kit (Omega).

### Fluorescence microscopy

Donor dsDNA and ssDNA were separately transfected into HEK293T cells. dsDNA was prepared from a purified PCR product that was dissolved in ddH_2_O. ssDNA was prepared by denaturation of dsDNA at 95 °C for 10 min in 100 mM NaCl. Transfected cells were photographed 24 h after transfection under a fluorescence microscope (NIKON).

### Cell culture and FACS

HEK293T cells were obtained from the American Type Culture Collection. Cells were cultured in DMEM (Dulbecco’s modified Eagle’s medium) containing 10% fetal bovine serum at 37 °C and 5% CO_2_. Fifteen micrograms of LentiCRISPR-V2 and 2 µg of donor DNA were cotransfected into 5 × 10^7^ cells with Lipofectamine 2000™ (Invitrogen) according to the manufacturer’s instructions. Subsequently, transfected cells were treated with 1 µg/mL puromycin for 72 h. Cells were washed with PBS and treated with 0.05% trypsin. The cell suspension was filtered through a 40 µm cell strainer (BD Falcon) before FACS. Flow cytometry analysis and FACS were performed using BD LSR II. Cells isolated by FACS were then cultured for one week and processed by FACS again to enhance the positive rate. Harvested cells were seeded in 96-well plates at 1/2 cell per well for single-clone growth.

### Genomic DNA extraction and analysis

Genomic DNA was extracted using the TIANamp Genomic DNA Kit (#DP304-03). PCR of single clones derived from genomic DNA was processed to verify that the clone possessed the desired sequence. PCR was performed for 35 cycles on an Eppendorf thermocycler with denaturation at 94 °C for 15 s, annealing at 58 °C for 15 s, and extension at 72 °C for 30 s using PrimeStar. Forward primers were located outside the left HA on the genome, and the universal reverse primer was located in the DsRed cassette.

The primers used were as follows:

RELA-gTest-F: GCTCATTGCCAAGGTGGGTA.

NABP2-gTest-F: GGATGGACCGAGTCCCGGCT.

EGFR-gTest-F: ATAAGAAGTCTGCAGAACTT.

Red Uni-R: TTGGACATGACTCCACAT.

### Conversion tract length detection

Multiple DNA variations on the left HA for *NABP2, RELA* and *EGFR* GEIS were introduced to HEK293T cells. FACS-sorted cells were collected for genomic DNA extraction. PCR was performed to amplify the successfully integrated DNA fragment from the donor DNA. PCR was performed for 28 cycles on an Eppendorf thermocycler with denaturation at 94 °C for 15 s, annealing at 58 °C for 15 s, and extension at 72 °C for 1 min using PrimeStar. PCR products were cloned into the vector pLV-MCS-puro-Green (digested by EcoRI) using Gibson assembly. A total of *E. coli* 624 colonies were sequenced. The mutations identified in the colonies were mapped to the wild-type NABP2 genomic sequence, and the seven candidate DNA alterations were recorded and calculated. The percentage of every variation was calculated as the number of mutated clones divided by 624 (total number of clones) and is illustrated in Fig. 2B.

The primers used were as follows:

EcoRI + NABP2-F: TTCTAGAGCTAGCGAATTGGATGGACCGAGTCCCGGCT.

EcoRI + Uni-R: CCGATTTAAATTCGAATTTTGGACATGACTCCACAT.

### Human and mouse exon length distribution

Human (GRCh38, release 37) and mouse (GRCm38, release M25) genomic annotation files from GENCODE were used to evaluate the distribution of CDSs and exon lengths. In brief, each exon (UTR contained or excluded) was identified, and its length was calculated based on the end and start positions in the genome. The calculated lengths were then grouped and illustrated.

### RNA extraction, RT–PCR and qPCR

Total RNA was extracted using a MolPure Cell/Tissue Total RNA Kit (Yeasen, China). RNA concentration was quantified by Nanodrop^C^ (Thermo, US). cDNA was processed with DNase treatment and reverse transcription from 500 ng total RNA using the Hifair III 1st Strand cDNA Synthesis Kit (Yeasen). Reverse transcription was performed on a thermocycler at 25 °C for 5 min, 55 °C for 15 min and then 85 °C for 5 min. RT–PCR was processed for 35 cycles on an Eppendorf thermocycler with denaturation at 94 °C for 15 s, annealing at 58 °C for 15 s, and extension at 72 °C for 20 s using PrimeStar. RT–PCR primers were set on exons adjacent to the processed intron to determine whether any alternative variants were produced. qPCR was performed to check whether there were any significant differences in expression in edited cells. qPCRs were processed using Hieff UNICON Universal Blue qPCR SYBR Green Master Mix (Yeasen) on QuantStudio Dx (ABI), and qPCR primer sequences were derived from PrimerBank. The qPCR was repeated three times.

The RT–PCR and qPCR primers were as follows:

RT-RELA-F: CTCGGTGGGGATGAGATCTT
RT-RELA-R: TTCTTCATGATGCTCTTGAA
RT-NABP2-F: GACAAAACAGGCAGCATCAA
RT-NABP2-R: GGGTTTGGCTCACTGAAGTT
RT-EGFR-F: GTGATGGCCAGCGTGGACAA
RT-EGFR-R: GGGATTCCGTCATATGGCTT
qGAPDH-F: GGAGCGAGATCCCTCCAAAAT
qGAPDH-R: GGCTGTTGTCATACTTCTCATGG

(product length = 197 bp)

qNABP2-F: TCTGGGACGATGTTGGCAAT
qNABP2-R: GGTGCCTGCTGGGTGCTGTA

(product length = 202 bp)

qRELA-F: CCCAACACTGCCGAGCTCAA
qRELA-R: CCTTTTACGTTTCTCCTCAA

(product length = 348 bp)

qEGFR-F: AGGCACGAGTAACAAGCTCAC
qEGFR-R: ATGAGGACATAACCAGCCACC

(product length = 177 bp)

## Supplementary Information

Below is the link to the electronic supplementary material.

Supplementary file1 (JPG 3411 KB)

Supplementary file2 (JPG 2680 KB)

Supplementary file3 (DOCX 508 KB)

Supplementary file4 (RAR 511 KB)

## Data Availability

Python script used in this study were online: https://github.com/wukaiyeah/Genomic_CDS_stats.git. FACS data can be downloaded from http://flowrepository.org/id/FR-FCM-Z4PY. Sanger sequencing data for conversion tract tests were submitted to GenBank as MZ399804—MZ400408. Sanger sequencing data for conversion tract tests are available in the online version of this manuscript (Supplementary Sanger sequencing data). Sanger sequencing data for conversion tract tests on *EGFR* and *RELA* were submitted as attached files. Sanger sequencing data for *NABP2* conversion tract tests were submitted to GenBank as MZ399804—MZ400408.
