# Supplementary figures and images for: Efficient gene editing through an intronic selection marker in cells
Source: Cell Mol Life Sci. 2022 Jan 31;79(2):111. doi: 10.1007/s00018-022-04152-1 (PMC8801403; doi:10.1007/s00018-022-04152-1)

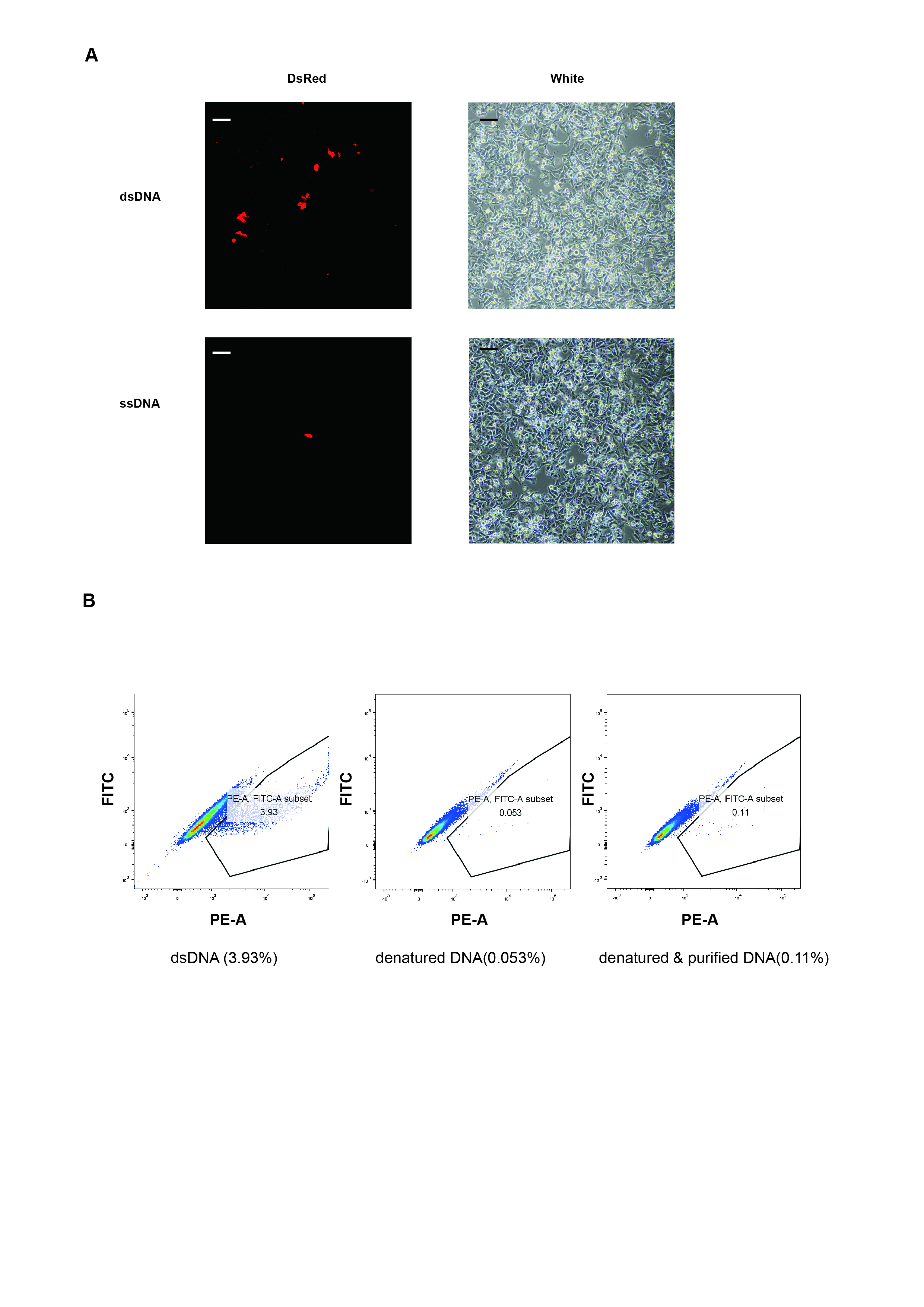

Supplement: Supplementary file 1 — Supplementary file1 (JPG 3411 KB) [file 18_2022_4152_MOESM1_ESM.jpg]

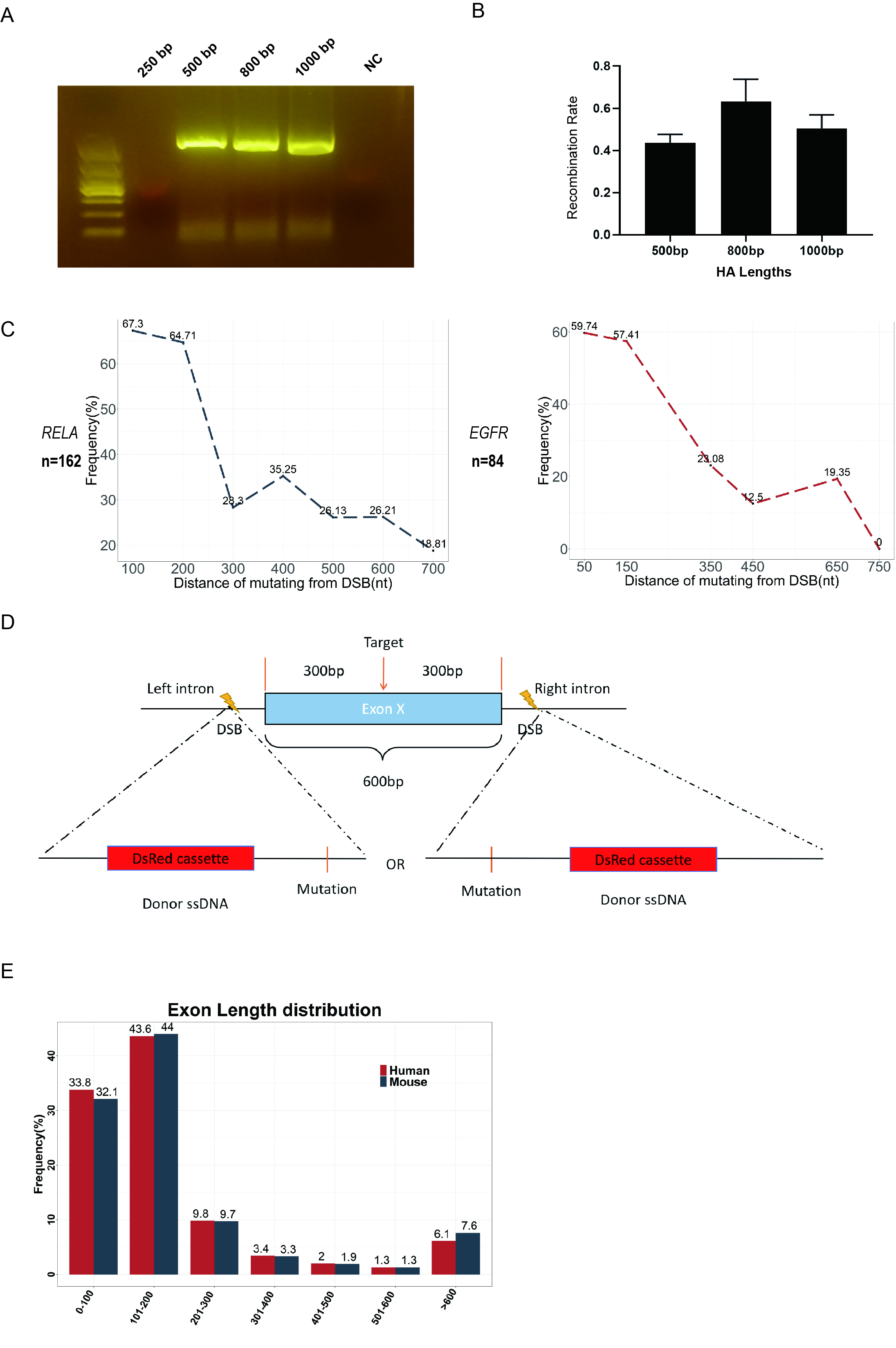

Supplement: Supplementary file 2 — Supplementary file2 (JPG 2680 KB) [file 18_2022_4152_MOESM2_ESM.jpg]
